# CEACAM1 Is a Prognostic Biomarker and Correlated with Immune Cell Infiltration in Clear Cell Renal Cell Carcinoma

**DOI:** 10.1155/2023/3606362

**Published:** 2023-01-18

**Authors:** Lu Yang, Yun Liu, Boke Zhang, Mengsi Yu, Fen Huang, Jiangzheng Zeng, Yanda Lu, Changcheng Yang

**Affiliations:** ^1^Department of Medical Oncology, The First Affiliated Hospital of Hainan Medical University, Haikou, Hainan 570102, China; ^2^Clinical Laboratory Center, The First Affiliated Hospital of Anhui University of Chinese Medicine, Hefei, Anhui 230031, China; ^3^Department of Clinical Laboratory, The First Affiliated Hospital of Xinjiang Medical University, Urumqi, Xinjiang 830054, China

## Abstract

**Background:**

CEACAM1 has been shown to be aberrantly expressed in a variety of tumors, and modulation of CEACAM1-related signaling pathways has been suggested as a novel approach for cancer immunotherapy in recent years. However, its role in clear cell renal cell carcinoma (ccRCC) is unclear.

**Methods:**

The relationship between CEACAM1 and ccRCC was demonstrated based on data from TCGA, GEO, and HPA databases. And the relationship between clinicopathological features and CEACAM1 expression was also assessed. Survival curve analysis was performed to analyze the prognostic relationship between CEACAM1 expression and ccRCC. Protein interaction network analysis was used to analyze the relationship between CEACAM1 and microenvironment-related proteins. In addition, the immunomodulatory role of CEACAM1 in ccRCC was assessed by analyzing CEACAM1 and immune cell infiltration.

**Results:**

The expression of CEACAM1 was lower in ccRCC tissues than in adjacent normal tissues, and its expression level was negatively correlated with tumor size status (*P* < 0.001), metastasis status (*P* = 0.009), pathological stage (*P* = 0.002), gender (*P* < 0.001), histological grade (*P* < 0.001), and primary therapy outcome (*P* = 0.045) of ccRCC. Survival curve analysis showed that ccRCC patients with lower CEACAM1 expression exhibited shorter overall survival (*P* < 0.001), and CEACAM1 interacted with microenvironmental molecules such as fibronectin and integrins. Furthermore, immune infiltration analysis showed that CEACAM1 expression correlated with CD8^+^ and CD4^+^ T cells, macrophage, neutrophil, and dendritic cell infiltration in ccRCC.

**Conclusions:**

CEACAM1 expression correlates with progression, prognosis, and immune cell infiltration in ccRCC patients, and it may be a promising prognostic biomarker and therapeutic target for ccRCC.

## 1. Background

In recent years, the incidence of renal cell carcinoma (RCC) has been increasing and has become one of the most common types of primary malignancy in adults [1]. Renal cell carcinoma is a heterogeneous cancer, with clear cell RCC (ccRCC) being the predominant type, accounting for approximately 80% of RCC [2]. ccRCC lacks effective treatment, and although surgery is the mainstay of treatment for ccRCC, approximately 35% of patients have already developed metastases when diagnosed, resulting in a high mortality rate of approximately 60% for ccRCC [3, 4]. Currently, targeted therapy is one of the standard treatments for ccRCC, but most patients eventually deteriorate due to drug resistance. Studies have confirmed that immunomodulation plays an important role in cancer progression, and thus, immunotherapy has been a major driving force for individualized cancer medicine [5]. Immune cell infiltration in the tumor microenvironment plays an important role in tumor progression [6]. However, the mechanisms of immune cell infiltration into the tumor microenvironment of ccRCC are still not fully elucidated.

As a member of the carcinoembryonic antigen family, carcinoembryonic antigen-associated cell adhesion molecule 1 (CEACAM1) is a single-channel transmembrane glycoprotein that was firstly identified in bile with initial appearance during embryonic development [7]. CEACAM1 is expressed not only in hepatocyte membranes but also in a variety of cells including epithelial cells, endothelial cells, and lymphocytes [8, 9]. Currently, studies have confirmed that CEACAM1 plays an important role in various biological functions such as apoptosis, angiogenesis, cell proliferation, invasion, migration, and fibrosis [7, 10]. When the ligand binds to CEACAM1, CEACAM1 transduces extracellular signals to the cell membrane region, so that the cytoplasmic tail of CEACAM1 causes intracellular signaling [11]. As a member of the TIM gene family, T cell immunoglobulin and mucin structural domain 3 (TIM-3) is mainly expressed in Th1, Th17, CD4^+^, and CD8^+^ T lymphocytes, etc., in which binding to ligands can inhibit the corresponding T lymphocyte response and induce immune tolerance [12, 13]. In recent years, it was found that the binding of CEACAM1 to TIM-3 mediates the function of suppressing T cells in autoimmunity and antitumor immunity [14, 15]. Studies have confirmed the presence of a large number of immune cell infiltrates in the tumor microenvironment of RCC, such as monocytes, NK cells, CD4^+^ and CD8^+^ T lymphocytes, and plasma cells [16, 17]. The altered infiltration pattern of immune cells in the microenvironment is closely related to the progression of renal cell carcinoma. For example, the levels of regulatory T cells and M2-type macrophages were significantly higher in high-risk patients compared to low-risk patients with ccRCC [18]. Since TIM-3, which binds to CEACAM1, are expressed in immune cells such as CD4^+^ and CD8^+^ T lymphocytes [13], CEACAM1 may play an important role in mediating immune cell infiltration in renal cell carcinoma. Therefore, understanding the relationship between CEACAM1 and tumor immune cell infiltration is of great importance, and CEACAM1 may become an attractive target for immunotherapy in renal cell carcinoma.

In this study, we downloaded and analyzed data from TCGA, GEO, and HPA databases to compare the differences of CEACAM1 expression between ccRCC tissues and normal samples adjacent to the tumor mass and to investigate the correlation between CEACAM1 expression levels and clinicopathological features. We then assessed the prognostic value of CEACAM1 by analyzing its association with the overall survival of ccRCC patients. In addition, we explored the correlation between CEACAM1 and other proteins to assess the possible action network of CEACAM1. Finally, we analyzed the correlation between CEACAM1 expression and immune cell infiltration of ccRCC. Our data suggested that CEACAM1 may be a promising prognostic biomarker and potential therapeutic target for the treatment of ccRCC.

## 2. Methods

### 2.1. Database and Data Acquisition

The Cancer Genome Atlas (TCGA) (https://genomecancer.ucsc.edu/) can provide clinical and pathological information on 33 types of cancer. Data on ccRCC patients and matching clinicopathological information are obtained through TCGA tool Cancer Browser. The Gene Expression Omnibus (GEO) database (https://www.ncbi.nlm.nih.gov/geo/) provides information on different normal tissues and the most typical cancers. Protein immunohistochemistry in normal human tissues and tumors is available from the Human Protein Atlas (HPA) database (https://www.proteinatlas.org). Since these databases are open and accessible, approval from the local ethics committee is not required.

### 2.2. Survival Analysis

According to the expression level of CEACAM1 gene, ccRCC patients were divided into a CEACAM1 high-expression group and CEACAM1 low-expression group. To investigate whether CEACAM1 expression levels affect the clinical prognosis of ccRCC patients, we used Kaplan-Meier (KM) survival curves to compare and analyze the differences in survival rates between the two groups.

### 2.3. Univariate and Multivariate Logistic Regression Analysis

To further determine the effect of CEACAM1 expression on the prognosis of ccRCC patients, we used the univariate Cox regression analysis to calculate the degree of association between the expression level of CEACAM1 and patients' overall survival. Then, multivariate analysis was used to assess whether CEACAM1 was an independent prognostic factor for overall survival in ccRCC patients. Factors were considered statistically significant in the Cox regression analysis when the *P* value was less than 0.05.

### 2.4. Protein-Protein Interaction Analysis

We used the online tool (STRING) website (https://string-db.org/) to retrieve interacting genes or proteins. CEACAM1 was imported into the online tool STRING to acquire the protein-protein interaction (PPI) network information. A confidence score greater than 0.7 was considered statistically significant.

### 2.5. TIMER Database Analysis

The Tumor Immune Estimation Resource (TIMER) from TCGA database is a public website covering 32 cancer types and containing 10,897 samples. This website enables the assessment of the abundance of immune cell infiltrates; therefore, the correlation between CEACAM1 expression and the abundance of six types of infiltrating immune cells (CD8^+^ T cells, CD4^+^ T cells, B cells, dendritic cells, macrophages, and neutrophils) was assessed in this website; thus, the relationship between CEACAM1 gene expression and tumor immune status was shown.

## 3. Results

### 3.1. The Clinical Characteristics of Patients

As shown in Table 1, the clinical information and gene expression data of 539 ccRCC patients were obtained from TCGA database. The gene expression data in FPKM format were converted into transcripts per million (TPM), which were then transformed by log2 for further analysis. The median expression level (log2) of CEACAM1 mRNA in ccRCC was 3.578 (range: 0.3594-6.051). Based on the cut-off value (3.578) of the relative CEACAM1 expression, ccRCC patients were divided into a high-expression group (*n* = 270) (>3.578) and a low-expression group (*n* = 269) (<3.578). In our study, the relationship between CEACAM1 expression level and clinicopathological characteristics of ccRCC patients was evaluated, and the results of chi-square test or Fisher's exact test have shown that CEACAM1 expression was negatively correlated with tumor size status (*P* < 0.001), metastasis status (*P* = 0.009), pathological stage (*P* = 0.002), and histological grade (*P* < 0.001) but positively correlated with primary treatment outcome (*P* = 0.045), and CEACAM1 expression was higher in male patients than in female patients. Moreover, the association between clinicopathological characteristics and CEACAM1 expression level was confirmed by logistic regression method. And the results also showed that CEACAM1 expression was associated with tumor size status (*P* = 0.002), metastasis status (*P* = 0.007), histologic grade (*P* < 0.001), pathologic stage (*P* = 0.001), and gender (*P* < 0.001) (Table 2).

### 3.2. The Expression of CEACAM1 in ccRCC Was Lower Than That in Normal Tissues

**Figure 1:**
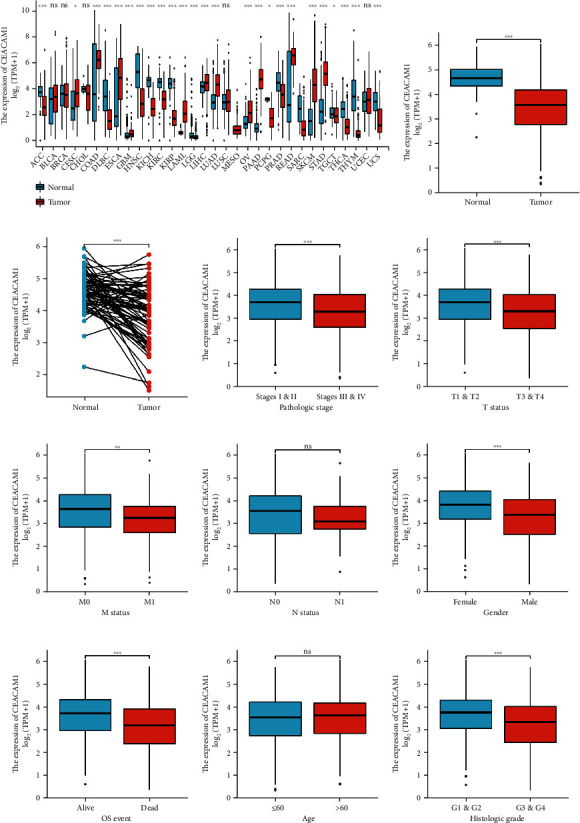
The expression status of CEACAM1 in cancers from TGCA database. (a) The expression levels of CEACAM1 in various cancer tissues and corresponding normal tissues. (b, c) Expression levels of CEACAM1 were significantly decreased in ccRCC tissues compared with normal tissues. (d–k) Lower expression of CEACAM1 was associated with pathological stage, T status, M status, gender, OS event, histological grade, and ccRCC classification; however, there was no statistically significant difference between CEACAM1 expression and pathological N status (g) and age (j). ^∗^*P* < 0.05, ^∗∗^*P* < 0.01, and ^∗∗∗^*P* < 0.001.

**Figure 2:**
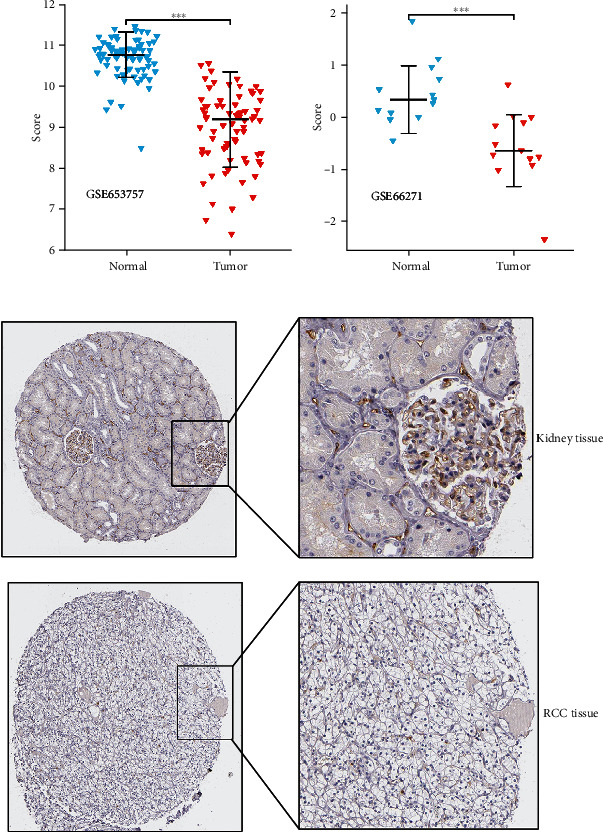
The gene expression of CEACAM1 in the GEO and HPA datasets. (a) Lower expression of CEACAM1 mRNA in ccRCC than paired normal tissues was verified in the GSE53757 dataset (*N* = 72). (b) Lower expression of CEACAM1 mRNA in ccRCC than in paired normal tissues was verified in the GSE66271 dataset (*N* = 13). (c) The protein levels of CEACAM1 in RCC tissues were lower than those in normal tissues in the Human Protein Atlas (antibody HPA065208, 10-fold). ^∗∗∗^*P* < 0.001.

The expression level of CEACAM1 mRNA was analyzed in different cancer types. In both TCGA and GEO databases, the gene expression level of CEACAM1 in ccRCC patient samples was significantly lower than that in adjacent normal tissues (Figures 1(a)–1(c) and 2(a) and 2(b)). And it was also verified in the immunohistochemical results of the HPA database; as shown in Figure 2(c), the expression of CEACAM1 was downregulated in ccRCC tissues compared to normal tissues. When analyzing the expression of CEACAM1 with clinicopathological parameters of ccRCC patients, the results showed no significant difference in CEACAM1 mRNA levels with age and lymph node status. However, lower CEACAM1 expression levels were observed in higher tumor size status, metastasis status, pathologic stage, histologic grade, unfavourable patient survival status, and male patients (Figures 1(d)–1(k)).

### 3.3. Lower Expression of CEACAM1 mRNA in ccRCC Showing Poor Prognosis

**Figure 3:**
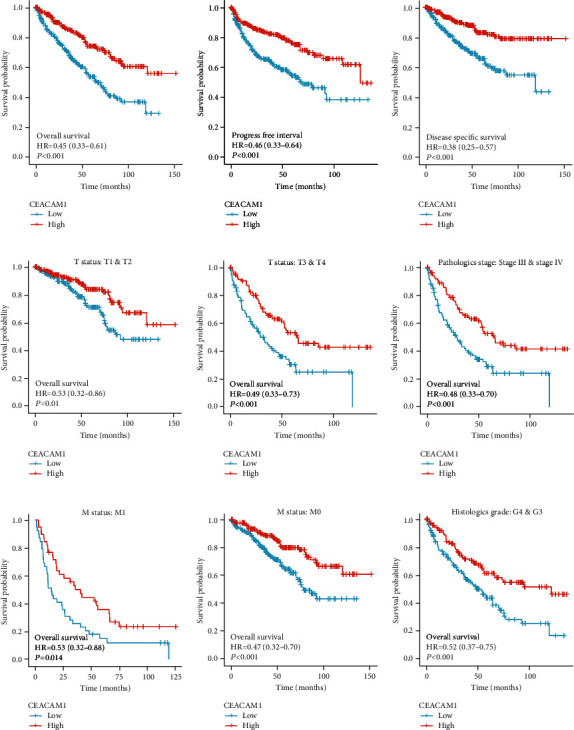
The Kaplan-Meier survival curves of the ccRCC patients with low and high CEACAM1 expression levels. (a–c) ccRCC patients with lower CEACAM1 expression exhibited shorter OS, PFI, and DSS (all *P* < 0.001). (d–i) ccRCC patients with lower CEACAM1 expression at high pathological grade, high histological grade, lymph node metastasis, and distant metastasis subgroups all exhibited shorter overall OS (all *P* < 0.05).

**Figure 4:**
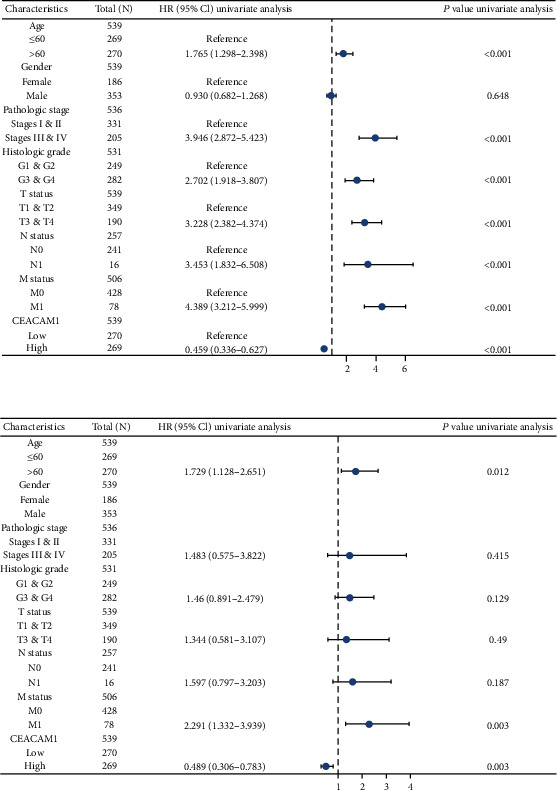
Univariate and multivariate regression analysis of CEACAM1 and other clinicopathological parameters with OS in ccRCC patients. (a) The results of the univariate Cox model showed that CEACAM1 expression and age, high pathological grade, and TNM stage were all predictors for OS in ccRCC patients (all *P* < 0.001). (b) CEACAM1 expression was an independent factor associated with OS in multivariate regression analysis (*P* = 0.003).

According to the KM plots based on TCGA database, ccRCC patients with lower CEACAM1 expression exhibited shorter overall survival (OS), progress-free interval (PFI), and disease-specific survival (DSS) (all *P* < 0.001) (Figures 3(a)–3(c)). In addition, ccRCC patients with lower CEACAM1 expression at high pathological grade, high histological grade, lymph node metastasis, and distant metastasis subgroups all showed shorter OS (all *P* < 0.05) (Figures 3(d)–3(i)). In the univariate Cox model, CEACAM1 expression and age, high pathological grade, and TNM stage were all significant predictors for OS in ccRCC patients (Table 3 and Figure 4(a)). And by multivariate regression analysis, CEACAM1 expression was also an independent factor associated with OS (Table 3 and Figure 4(b)).

### 3.4. Building Protein Interaction Networks

**Figure 5:**
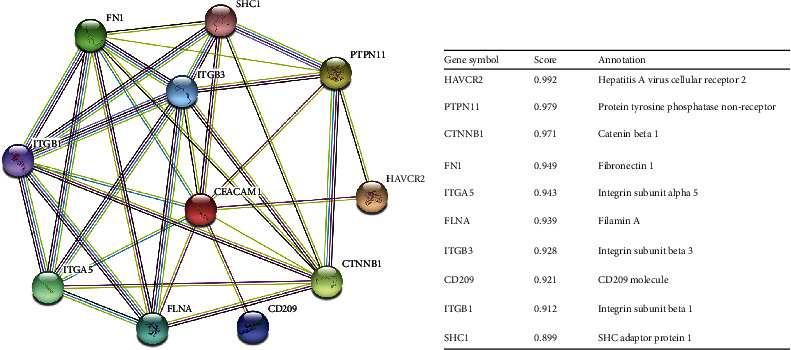
(a) CEACAM1-interacting proteins in ccRCC tissues. (b) Annotation of CEACAM1-interacting proteins and their coexpression scores.

Functional interactions between protein molecules are necessary for malignancy progression and its molecular mechanisms. Thus, we analyzed the PPI network of CEACAM1 proteins using the STRING tool to determine their role in ccRCC progression. The top 10 proteins and their corresponding gene names, scores, and gene annotations are listed in Figure 5. These genes include HAVCR2, PTPN11, CTNNB1, FN1, ITGA5, FLNA, ITGB3, CD209, ITGB1, and SHC1.

### 3.5. Correlation between CEACAM1 Expression and Immune Cell Infiltration

**Figure 6:**
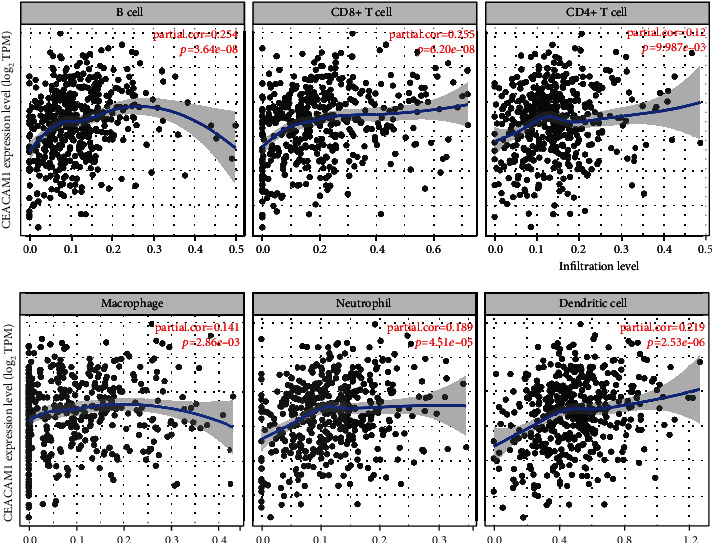
Association of CEACAM1 expression with infiltrating immune infiltration in ccRCC. The expression levels of CEACAM1 had a significant positive correlation with the infiltration levels of B cells, CD8^+^ T cells, CD4^+^ T cells, macrophages, neutrophil granulocytes, and dendritic cells.

Immune cell infiltration in tumor tissue affects the survival of cancer patients. Therefore, we analyzed the correlation between CEACAM1 expression and six types of infiltrating immune cells including B lymphocytes, CD8^+^ T cells, CD4^+^ T cells, macrophages, neutrophils, and dendritic cells. The results showed that the expression level of CEACAM1 had a significant positive correlation with the infiltration levels of B cells (*r* = 0.254, *P* = 3.64*e* − 08), CD8^+^ T cells (*r* = 0.255, *P* = 6.20*e* − 08), CD4^+^ T cells (*r* = 0.120, *P* = 9.87*e* − 03), macrophages (*r* = 0.141, *P* = 2.86*e* − 03), neutrophil granulocytes (*r* = 0.189, *P* = 4.51*e* − 05), and dendritic cells (*r* = 0.219, *P* = 2.53*e* − 06) (Figure 6).

## 4. Discussion

Currently, whether CEACAM1 promotes or inhibits tumor progression has been controversial. For example, CEACAM1 was found to promote the invasion and progression of melanoma, pancreatic cancer, non-small-cell lung cancer, etc. [10, 19–21]. Silencing CEACAM1 by siRNA can inhibit the invasion and migration of glioma [22], which suggests that inhibition of CEACAM1 could suppress tumor progression. However, our previous study and other groups' studies have suggested that CEACAM1 is a cancer suppressor molecule that inhibits the development of tumor. For instance, CEACAM1 may play an anticancer role in breast cancer and prostate cancer, and animal experiments have also demonstrated that breast cancer cells or prostate cancer cells overexpressing CEACAM1 have a reduced tumorigenic capacity [23–25]. In addition, CEACAM1 expression was significantly reduced in breast and prostate cancers and predicts a poor prognosis for these cancers [26, 27]. In the present study, we investigated the expression of CEACAM1 in the development and progression of ccRCC and its prognosis. Our study revealed that CEACAM1 downregulation was associated with tumorigenesis and progression of ccRCC. We found that the gene expression level of CEACAM1 in ccRCC samples was significantly lower than that in nontumor normal tissues, which was consistent with previous studies [28]. Therefore, CEACAM1 may act as a tumor suppressor for ccRCC, and its reduced expression in tumor tissues contributes to tumor progression. Generally, the downregulation of antioncogene expression may be due to gene deletion or mutation, transcriptional repression, translational repression, or increased degradation. Based on current knowledge, CEACAM1 expression is regulated mainly at the transcriptional level [29, 30]. For example, the transcription factor Sp2 may repress CEACAM1 gene expression by recruiting histone deacetylase activity to the CEACAM1 promoter [29]. However, in ccRCC, there is no evidence to confirm the specific mechanism of CEACAM1 downregulation and further studies are needed in the future. Furthermore, the expression level of CEACAM1 was also negatively related to tumor size status, histological grade, and pathological stage. In addition, ccRCC patients with low expression of CEACAM1 had a poorer prognosis compared to patients with high CEACAM1 expression. Therefore, these findings further suggest that CEACAM1 plays an inhibitory role in the progression of ccRCC.

As a cell membrane protein, extracellular matrices fibronectin and integrin have been identified as important proteins interacting with CEACAM1 [31, 32]. Therefore, it is likely that CEACAM1 promotes invasion and progression by interacting with related molecules in the tumor microenvironment. Interestingly, earlier studies have found that CEACAM1 is involved in neovascularization by affecting endothelial cytoskeleton structure and integrin-mediated signaling [31]. In addition, CEACAM1 could alter integrin affinity, which in turn enhances neutrophil adhesion to fibronectin [32]. In our study, according to the analysis of STING software, CEACAM1 are also considered as important proteins interacting with fibronectin 1 (FN1) and integrin subunit alpha 5 (ITGA5) in ccRCC. However, the biologic role of the interaction between CEACAM1 and molecules such as fibronectin and integrins in ccRCC remains unclear and further studies are needed.

CEACAM1 is expressed on various immune cells, tumor cells, and other cells to exert various biological functions. CEACAM1 has been reported to mediate antitumor effects in vivo, and targeting the CEACAM1-related signaling pathway has recently been considered as a new approach for cancer immunotherapy [33]. Our study found that CEACAM1 expression correlated with CD8^+^ T cells, CD4^+^ T cells, macrophages, neutrophils, and dendritic cell infiltration in ccRCC. CD8^+^ T cells are known to be the main effector cells in cancer immunotherapy. Activated CD8^+^ T cells suppress tumors mainly through stimulation of programmed cell death via the Fas-Fas ligand pathway and release of perforin [34]. There is one study showing the coexpression of CEACAM1 and TIM-3 molecules on CD8^+^ T cell in a mouse colorectal cancer model and human colorectal cancer patients, and CEACAM1 can be upregulated by IFN-*γ* and interleukin-27, leading to the suggestion that CEACAM1 may be a phenotype of failing T cells [15]. Other studies have also confirmed the involvement of the TIM3/CEACAM1 pathway in the depletion of CD8^+^ T cells, further suggesting that CEACAM1 is an important regulator of CD8^+^ T cell function [35]. However, some studies have found that tumor-infiltrating CD8^+^ T cells expressing low level of CEACAM1 have no or minimal antitumor effects [36]. RCC is one of the most immunoinfiltrative tumors, and it has been found that the infiltrating immune cells mainly include cytotoxic T cells (CD8^+^ T cells), which are associated with a poor prognosis [37]. In clinical practice, immunotherapy is emerging as a treatment for RCC; for example, PD-1/PD-L1 antibody therapy has been approved as a first-line treatment for metastatic RCC. Therefore, the prognosis of patients can be predicted by further studying the tumor immune microenvironment in ccRCC.

Whether CEACAM1 positively or negatively regulates other immune cells is also very complex, and its regulatory mechanism is not well understood. There is a study suggesting that CEACAM1, particularly the CEACAM1-3S isoform, was able to induce enhanced expression of the NKG2D ligands MICA and ULBP2 on the surface of melanoma cells, which in turn contributed to NK cell-mediated cytolysis of tumor cells [38]. CEACAM1 also plays a key role in the development and function of monocyte or macrophages. For example, CEACAM1 was found to promote mouse monocyte survival by inhibiting mitochondrion-mediated apoptosis in mouse monocytes [39]. CEACAM1 is rapidly transferred from the cytoplasm to the membrane to become a cellular receptor for bacterial pathogens exerting a negative neutrophil regulatory effect [40]. Moreover, CEACAM1 is the only member of the CEACAM family expressed on activated T cells. A small number of CD4^+^ T cells expressing CEACAM1 can be detected in peripheral blood [41], but their identity and function are not yet clear.

The limitations of this study are mainly due to biases caused by differences in database data, analysis platforms, and different grading and staging criteria. In addition, the group ethnology information in TCGA database was mainly limited to individual ethnic group, while other ethnic groups were not studied. The results analyzed lacked specific experimental method validation and functional analysis of CEACAM1. Further studies are needed to analyze CEACAM1-based immunotherapy in ccRCC in vitro and in vivo.

## 5. Conclusion

In summary, we observed downregulated expression of CEACAM1 in ccRCC, which is also associated with poor prognosis of ccRCC patients. Furthermore, CEACAM1 may be involved in the progression of ccRCC by affecting the function of immune infiltrating cells. The current study partially reveals the role of CEACAM1 in immunotherapy and provides a potential prognostic biomarker and immunotherapy target for ccRCC.

## Figures and Tables

**Table 1:** Clinical characteristics of the ccRCC patients in the study.

Characteristics	Low expression of CEACAM1 (<3.578)	High expression of CEACAM1 (>3.578)	*P* value
*n*	269	270	
T status, *n* (%)			<0.001
T1	117 (21.7%)	161 (29.9%)	
T2	40 (7.4%)	31 (5.8%)	
T3	102 (18.9%)	77 (14.3%)	
T4	10 (1.9%)	1 (0.2%)	
N status, *n* (%)			0.293
N0	125 (48.6%)	116 (45.1%)	
N1	11 (4.3%)	5 (1.9%)	
M status, *n* (%)			0.009
M0	202 (39.9%)	226 (44.7%)	
M1	50 (9.9%)	28 (5.5%)	
Pathologic stage, *n* (%)			0.002
Stage I	115 (21.5%)	157 (29.3%)	
Stage II	32 (6%)	27 (5%)	
Stage III	68 (12.7%)	55 (10.3%)	
Stage IV	53 (9.9%)	29 (5.4%)	
Gender, *n* (%)			<0.001
Female	70 (13%)	116 (21.5%)	
Male	199 (36.9%)	154 (28.6%)	
Age, *n* (%)			0.464
≤60	139 (25.8%)	130 (24.1%)	
>60	130 (24.1%)	140 (26%)	
Histologic grade, *n* (%)			<0.001
G1	3 (0.6%)	11 (2.1%)	
G2	101 (19%)	134 (25.2%)	
G3	112 (21.1%)	95 (17.9%)	
G4	50 (9.4%)	25 (4.7%)	
Laterality, *n* (%)			0.545
Left	130 (24.2%)	122 (22.7%)	
Right	139 (25.8%)	147 (27.3%)	
Primary therapy outcome, *n* (%)			0.045
PD	9 (6.1%)	2 (1.4%)	
SD	2 (1.4%)	4 (2.7%)	
PR	0 (0%)	2 (1.4%)	
CR	58 (39.5%)	70 (47.6%)	
Age, mean ± SD	60.46 ± 11.95	60.8 ± 12.26	0.745

T: tumor size; N: lymph node; M: metastasis; PD: progressive disease; SD: stable disease; PR: partial response; CR: complete response.

**Table 2:** Logistic regression analysis of the association between CEACAM1 expression and clinicopathological features in ccRCC patients.

Characteristics	Total (*N*)	Odds ratio (OR)	*P* value
T status (T3 and T4 vs. T1 and T2)	539	0.569 (0.397-0.813)	0.002
N status (N1 vs. N0)	257	0.490 (0.151-1.390)	0.198
M status (M1 vs. M0)	506	0.501 (0.300-0.820)	0.007
Age (>60 vs. ≤60)	539	1.151 (0.821-1.615)	0.413
Histologic grade (G3 and G4 vs. G1 and G2)	531	0.531 (0.376-0.749)	<0.001
Pathologic stage (stage III and stage IV vs. stage I and stage II)	536	0.555 (0.389-0.788)	0.001
Primary therapy outcome (PD and SD vs. CR and PR)	147	0.439 (0.144-1.227)	0.126
Gender (male vs. female)	539	0.467 (0.324-0.670)	<0.001
Laterality (right vs. left)	538	1.127 (0.803-1.582)	0.490

T: tumor size; N: lymph node; M: metastasis; PD: progressive disease; SD: stable disease; PR: partial response; CR: complete response.

**Table 3:** Associations between overall survival and clinicopathological characteristics in ccRCC patients by Cox regression.

Characteristics	Total (*N*)	Univariate analysis		Multivariate analysis	
		Hazard ratio (95% CI)	*P* value	Hazard ratio (95% CI)	*P* value
Age	539				
≤60	269	Reference			
>60	270	1.765 (1.298-2.398)	<0.001	1.729 (1.128-2.651)	0.012
Gender	539				
Female	186	Reference			
Male	353	0.930 (0.682-1.268)	0.648		
Pathologic stage	536				
Stage I and stage II	331	Reference			
Stage III and stage IV	205	3.946 (2.872-5.423)	<0.001	1.483 (0.575-3.822)	0.415
Histologic grade	531				
G1 and G2	249	Reference			
G3 and G4	282	2.702 (1.918-3.807)	<0.001	1.486 (0.891-2.479)	0.129
T status	539				
T1 and T2	349	Reference			
T3 and T4	190	3.228 (2.382-4.374)	<0.001	1.344 (0.581-3.107)	0.490
N status	257				
N0	241	Reference			
N1	16	3.453 (1.832-6.508)	<0.001	1.597 (0.797-3.203)	0.187
M status	506				
M0	428	Reference			
M1	78	4.389 (3.212-5.999)	<0.001	2.291 (1.332-3.939)	0.003
CEACAM1	539				
Low	270	Reference			
High	269	0.459 (0.336-0.627)	<0.001	0.489 (0.306-0.783)	0.003

T: tumor size; N: lymph node; M: metastasis.

## Data Availability

The datasets analyzed in the current study are available in the GEO (https://www.ncbi.nlm.nih.gov/geo/), HPA (https://www.proteinatlas.org), and TCGA database (https://portal.gdc.cancer.gov/).

## Abbreviations

CEACAM1: Carcinoembryonic antigen-related cell adhesion molecule 1
ccRCC: Clear cell renal cell carcinoma
TCGA: The Cancer Genome Atlas
GEO: Gene Expression Omnibus
HPA: Human Protein Atlas
OS: Overall survival
PFI: Progress-free interval
DSS: Disease-specific survival
TIMER: Tumor Immune Estimation Resource.
